# Rubi Fructus (*Rubus coreanus*) Inhibits Differentiation to Adipocytes in 3T3-L1 Cells

**DOI:** 10.1155/2013/475386

**Published:** 2013-10-30

**Authors:** Mi-Young Jeong, Hye-Lin Kim, Jinbong Park, Hyo-Jin An, Sung-Hoon Kim, Su-Jin Kim, Hong-Seob So, Raekil Park, Jae-Young Um, Seung-Heon Hong

**Affiliations:** ^1^Department of Oriental Pharmacy, College of Pharmacy, Wonkwang-Oriental Medicines Research Institute, Wonkwang University, Jeonbuk 570-749, Republic of Korea; ^2^College of Korean Medicine, Institute of Korean Medicine, Kyung Hee University, 1 Hoegi-Dong, Dongdaemun-gu, Seoul 130-701, Republic of Korea; ^3^Department of Pharmacology, College of Oriental Medicine, Sangji University, Wonju-si, Gangwon-do 220-702, Republic of Korea; ^4^Department of Cosmeceutical Science, Daegu Hanny University, Yugok-dong, Kyungsan 712-715, Republic of Korea; ^5^Center for Metabolic Function Regulation, Wonkwang University, Iksan, Jeonbuk 570-749, Republic of Korea

## Abstract

Rubi Fructus (RF) is known to exert several pharmacological effects including antitumor, antioxidant, and anti-inflammatory activities. However, its antiobesity effect has not been reported yet. This study was focused on the antidifferentiation effect of RF extract on 3T3-L1 preadipocytes. When 3T3-L1 preadipocytes were differentiating into adipocytes, 10–100 **μ**g/mL of RF was added. Next, the lipid contents were quantified by Oil Red O staining. RF significantly reduced lipid accumulation and downregulated the expression of peroxisome proliferator-activated receptor **γ** (PPAR**γ**), CCAAT0-enhancer-binding proteins **α** (C/EBP**α**), adipocyte fatty acid-binding protein 2 (aP2), resistin, and adiponectin in ways that were concentration dependent. Moreover, RF markedly upregulated liver kinase B1 and AMP-activated protein kinase (AMPK). Interestingly, pretreatment with AMPK**α** siRNA and RF downregulated the expression of PPAR**γ** and C/EBP**α** protein as well as the adipocyte differentiation. Our study shows that RF is capable of inhibiting the differentiation of 3T3-L1 adipocytes through the modulation of PPAR**γ**, C/EBP**α**, and AMPK, suggesting that it has a potential for therapeutic application in the treatment or prevention of obesity.

## 1. Introduction

Obesity is associated with many diseases inducing diabetes, dyslipidemia, and atherosclerosis, which are risk factors for metabolic syndrome [1]. Obesity is a condition in which excess body fat has accumulated due to lipids changing into adipocytes and an increase in the number of differentiated mature cells which are regulated by genetic and environmental factors such as nutrients [2–4]. Understanding the mechanism through which a particular nutrient affects the differentiation to adipocytes would help to prevent the initiation and progression of obesity. The 3T3-L1 cell line is one of the best-characterized and reliable models for studying the conversion of preadipocytes into adipocytes. Adipocyte differentiation is a complex process involving coordinated expression of specific genes and proteins associated with each stage of adipogenesis [5, 6]. 

Peroxisome proliferator-activated receptor *γ* (PPAR*γ*) and CCAAT-enhancer-binding protein *α* (C/EBP*α*) have been known to play a key role in the regulation of adipogenesis and in the modulation of fat cell function in adipose tissue. PPAR*γ*, a member of the PPAR subfamily of nuclear hormone receptors, was identified as a component of a differentiation-dependent regulatory factor and a far-cell-specific enhancer of the adipocyte fatty acid-binding protein (aP2) gene [7, 8]. Another major protein in obesity regulation, C/EBP*α*, a member of a large family of leucine zipper transcription factors, plays an important role in induction of terminal adipocyte differentiation [9, 10]. PPAR*γ* and C/EBP*α* are not expressed in preadipocytes but are activated during adipocyte differentiation. PPAR*γ* and C/EBP*α* are expressed prior to the expression of most adipocyte genes and regulate the expression of genes involved in creating and maintaining adipocytes, including aP2 and resistin [11, 12]. 

AMP-activated protein kinase (AMPK) is a serine/threonine protein kinase that is widely expressed in eukaryotes. AMPK, composed of *α*, *β*, and *γ* subunits, is a key player in energy homeostasis. The *α* subunit is the catalytic subunit, and its activation via the phosphorylation of the threonine residue 172 by the upstream liver kinase B1 (LKB1) is crucial for AMPK activation under ATP-depleted conditions [13]. When the intracellular AMP/ATP ratio increases because of the metabolic stress, AMPK is phosphorylated. Subsequently, downstream target molecules are activated, promoting catabolism. When the intracellular AMP/ATP ratio decreases, AMPK increases the anabolism. AMPK is associated with adipocyte differentiation via AMPK activation in 3T3-L1 adipocytes [14, 15]. In addition, AMPK inhibits the accumulation of fat by modulating downstream substrate acetyl-CoA carboxylase (ACC) [16].

Rubi Fructus (RF), the fruit of *Rubus coreanus *Miquel (Rosaceae), is a type of red raspberry from southern Korea. The dried fruit are used in traditional herbal medicine for the treatment of impotence, spermatorrhea, enuresis, and asthma [17]. RF includes functional constituents that include flavonoids, anthocyanin, polyphenols, niga-ichigoside F1, 23-hydroxytormentic acid, and gallic acid [18, 19]. It has been found that these constituents show anticarcinogenic, antinociceptive, antioxidant, and anti-inflammatory effects [20, 21]. Even though numerous biological activities of RF have been reported, there is limited evidence for its antiobesity effect. In this study, we evaluated the inhibitory effect of the water extract of RF and investigated how it acts to reduce differentiation to adipocytes in 3T3-L1 mouse fibroblasts.

## 2. Materials and Methods

### 2.1. Preparation of RF

Dried and ground powder of RF, the fruit of *Rubus coreanus *Miquel (Rosaceae), was kindly provided by Kyung Hee Oriental Hospital (Seoul, Republic of Korea). To prepare the aqueous extract, the water-soluble components of the RF powder were extracted with water (100 g/L of water) by heating at 100°C for 3 h. The boiled solution was filtered through Whatman filter paper, and the filtrates were lyophilized. The resulting powder was used as the crude total extract of the fruit. Total extracts were dissolved in water for cell treatment. The crude extract (10 g) was subsequently partitioned between ethyl acetate (EtOAc) and distilled water. The EtOAc fraction was concentrated under reduced pressure in a rotary evaporator and lyophilized. The EtOAc fractions were dissolved with DMSO, filtered using sterilized syringe filter through a 0.22 *μ*m membrane pore, and stored at −20°C before use.

### 2.2. Reagents

Dulbecco's modified Eagle's medium (DMEM), penicillin-streptomycin, fetal calf serum (FCS), and fetal bovine serum (FBS) were from Gibco BRL (Grand Island, NY, USA). Insulin, 3-isobutylmethylxanthine (IBMX), and dexamethasone (DEX) were purchased from Sigma Chemical Co. (St. Louis, MO, USA). Anti-C/EBP*α*, anti-resistin, and anti-GAPDH antibodies were purchased from Santa Cruz Biotechnology (Santa Cruz, CA, USA). Anti-PPAR*γ*, anti-aP2, anti-adiponectin, anti-phospho LKB1, anti-phospho AMPK*α* anti-AMPK*α*, and anti-ACC antibodies were purchased from Cell Signaling Technology (Beverly, MA, USA). 

### 2.3. Cell Culture and Adipocyte Differentiation

3T3-L1 mouse embryo fibroblasts were obtained from the American Type Culture Collection (Rockville, MD, USA). Cells were grown in DMEM plus 10% calf serum and plated for final differentiation in DMEM plus 10% FBS with 100 units/mL of penicillin-streptomycin solution at 37°C, in 5% CO_2_, at 95% humidity until confluence. Two days after confluence (Day 0), the cells were stimulated to differentiate with differentiation inducers (1 *μ*M dexamethasone, 500 *μ*M 3-isobutyl-1-methylxanthine, and 1 *μ*g/mL insulin, MDI) that were added to DMEM containing 10% FBS for two days (Day 2). Preadipocytes were then cultured in DMEM, 10% FBS supplemented with 1 *μ*g/mL insulin for another two days (Day 4), followed by culturing with 10% FBS/DMEM medium for an additional two days (Day 6), at which time more than 90% of cells were mature adipocytes with accumulated fat droplets. On Day 2, RF was prepared in a differentiation medium at concentrations of 10 *μ*g/mL, 50 *μ*g/mL, and 100 *μ*g/mL. 

### 2.4. Cell Cytotoxicity Assay

Cell viability was measured with a CellTiter 96 Aqueous One Solution Cell Proliferation Assay Kit (Promega Corporation, Madison, USA) according to the manufacture's instruction. Briefly, The cells (5 × 10^3^ per 96 well) were incubated at 37°C in 5% CO_2_ and 95% air with different concentrations of RF. After 48 h for 3T3-L1 preadipocytes, 20 *μ*L of MTS [3-(4,5-dimethylthiazol-2-yl)-5-(3-carboxymethoxyphenyl)-2(4-sulfophenyl)-2H-tetrazolium, inner salt] solution was added to each well, incubated for 4 h, and absorbance at 490 nm was measured using a VERSAmax microplate reader (Molecular Devices, Sunnyvale, CA, USA) to determine the formazan concentration, which is proportional to the number of live cells.

### 2.5. Oil Red O Staining

Intracellular lipid accumulation was measured using Oil Red O. The Oil Red O working solution was prepared as described by Ramirez-Zacarias et al. [22]. The 3T3-L1 cells were washed twice with phosphate-buffered saline (PBS) and were then fixed in 10% formaldehyde in PBS for 1 h. After washing with 60% isopropanol, the cells were stained with Oil Red O solution for 30 min at room temperature. The cells were washed with water four times to remove the unbound dye. The stained cells were observed with an Olympus IX71 Research Inverted Phase microscope (Olympus Co., Tokyo, Japan). Following the microscopic observation, 100% isopropanol was added as an extraction solution to extract the excess staining dye from the cells. The absorbance of the extracted dye was measured spectrophotometrically at 500 nm in a VERSAmax microplate reader (Molecular Devices, Sunnyvale, CA, USA). 

### 2.6. RNA Isolation and Real-Time RT-PCR

Total RNA was extracted using a GeneAll RiboEx Total RNA extraction kit (GeneAll Biotechnology, Seoul, Republic of Korea) and QIAzol lysis reagent (QIAZEN sciences, Maryland, USA). RNA (2 *μ*g) was used as a template for first-strand cDNA synthesis performed using a Power cDNA Synthesis Kit (INTRON Biotechnology, Seoul, Republic of Korea) according to the manufacturer's instructions. Newly synthesized cDNA from 3T3-L1 control cells and RF-treated cells was amplified using specific primers and the Fast SYBR Green PCR Master Mix (Applied Biosystems, Foster City, CA, USA). PCR products were measured with a StepOnePlus Real-Time RT-PCR System, and the relative gene expression was calculated based on the comparative CT method using a StepOne software v2.1 (Applied Biosystems, Foster City, CA, USA). The expression of Glyceraldehyde-3-phosphate dehydrogenase (GAPDH) mRNA was used as an endogenous control. The primers used in the experiments are shown in Table 1.

### 2.7. Western Blot Analysis

3T3-L1 cells were harvested and washed with PBS and then collected by centrifugation at 13,000 rpm for 1 min at 4°C. To obtain the cellular lysate, cells were lysed on ice for 30 min in RIPA buffer (50 mM Tris-HCl, pH 7.5, 0.15 M NaCl, 1% NP-40, 0.1% sodium dodecyl sulfate (SDS), 1 mM DTT, and 1 mM PMSF), which contained a mixture of protease inhibitors (Sigma, Mannheim, Germany). Insoluble materials were removed by centrifugation at 13,000 rpm for 15 min at 4°C. A total of 20 *μ*g of the supernatants were separated using 10% sodium dodecyl sulfate-polyacrylamide gel electrophoresis (SDS-PAGE) and transferred to polyvinylidene difluoride (PVDF) membranes. After blocking with 10 mM Tris, 150 mM NaCl, and 0.05% Tween-20 (TBST) (pH 7.6) containing 5% skim milk for 1 h at room temperature, the membranes were washed with TBST. The membranes were incubated overnight with anti-PPAR*γ*, anti-pAMPK, anti-AMPK, anti-C/EBP*α*, and GAPDH at 4°C. After washing with TBST, the blots were subsequently incubated with horseradish peroxidase (HRP)-conjugated affinipure Goat anti-rabbit IgG or Goat anti-mouse IgG (Jackson ImmunoResearch Laboratory, USA) in 5% skim milk-TBST at room temperature for 1 h. Protein signals were developed using the ECL Western Blotting Detection Reagent (Amersham Bioscience, Piscataway, NJ, USA). All experiments were repeated at least three times. Representative Western blots are shown along with the graphs of the quantitative data. The chemiluminescent intensities of protein signals were quantified using Quantity One software (Bio-Rad Laboratories, Hercules, CA, USA). 

### 2.8. AMPKa siRNA Transfection

The transfection of siRNAs was performed according to the manufacturer's instructions. Preadipocytes were seeded in 6-well plates, and siRNA transfection was carried out 2 days after the confluence of preadipocytes. Lipofectamine 2000 (5 *μ*L) and 10 *μ*L siRNA (10 pmol) were individually diluted and incubated in 250 *μ*L Opti-MEM medium (Invitrogen, Carlsbad, CA, USA) for 20 min. Next, these were mixed and incubated for 30 min before being added to each well. The initial medium was removed and replaced with induction medium 48 h after transfection.

### 2.9. Statistical Analysis

Results are expressed as the mean ± SEM of independent experiments, and statistical analyses were performed using Student's *t-*test to determine differences between groups. All statistical analyses were performed using SPSS statistical analysis software version 11.5 (SPSS Inc., Chicago, IL, USA). Values with *P < 0.05 were considered to indicate statistical significance. 

## 3. Results

### 3.1. Effects of RF on Cytotoxicity and Inhibition of Adipogenesis in 3T3-L1 Adipocytes

**Figure 1:**
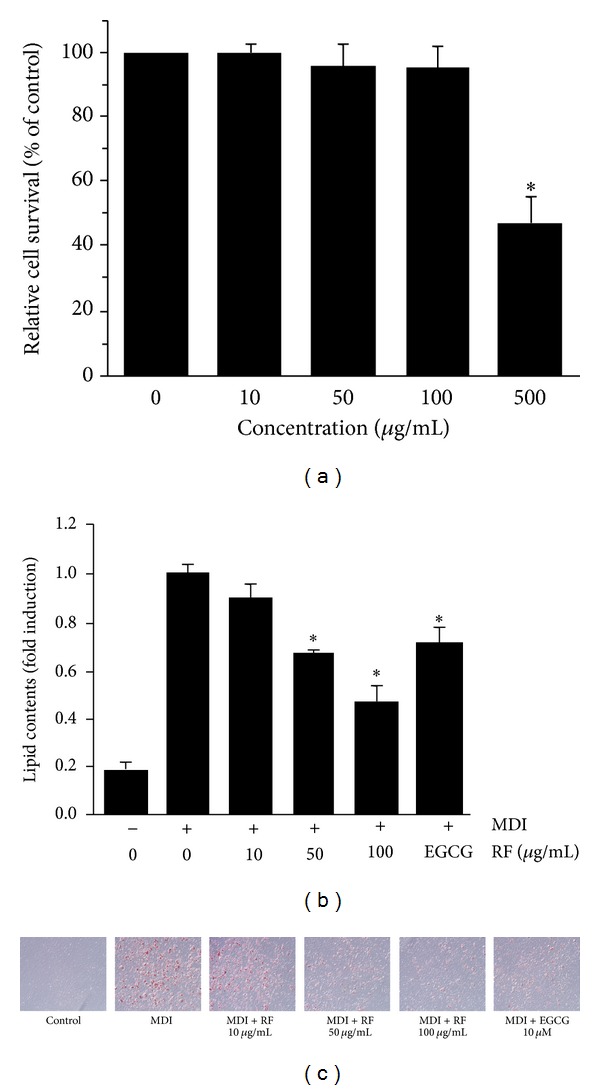
Effect of RF on cell viability and lipid accumulation in 3T3-L1 cells. (a) 3T3-L1 cells were treated with RF at various concentrations (10–500 *μ*g/mL) for 48 h. Cell viability was determined by the MTS assay. Postconfluent 3T3-L1 cells were differentiated in the absence or in the presence of RF (0, 10, 50, and 100 *μ*g/mL) for 6 days. (b) Triglyceride content was quantified by measuring absorbance. EGCG was used as positive control. Assays were performed in duplicate for each concentration, and experiments were repeated at least three times. Data are expressed as means ± S.D. where *P* < 0.05 was considered a statistically significant difference from the differentiated control. (c) Lipid droplets were measured by Oil Red O staining.

To determine the cytotoxicity of RF, 3T3-L1 cells were treated with various concentrations (10–500 *μ*g/mL) of RF, and the cell viability was measured by the MTS assay. As shown in Figure 1(a), treatment with 10–100 *μ*g/mL of RF did not cause significant cytotoxic effects on 3T3-L1 cells. Next, we measured the effect of RF on adipocyte differentiation. We used a differentiation mixture (MDI) to induce the differentiation of 3T3-L1 cells. The 3T3-L1 cells were treated with 10, 50, and 100 *μ*g/mL of RF during differentiation; after 6 days, cells were stained with Oil Red O. As shown in Figures 1(b) and 1(c), RF suppressed adipocyte differentiation in a dose dependent manner in 3T3-L1 cells, and adipogenesis was compared with the treatment of epigallocatechin gallate (EGCG), which is a known differentiation blocker. The cells treated with 50 and 100 *μ*g/mL of RF showed a significant reduction in lipid accumulation through the inhibition of differentiation of 3T3-L1 preadipocytes.

### 3.2. Comparative Effects of RF Extraction Fraction on Inhibition of Adipogenesis in 3T3-L1 Adipocytes

**Figure 2:**
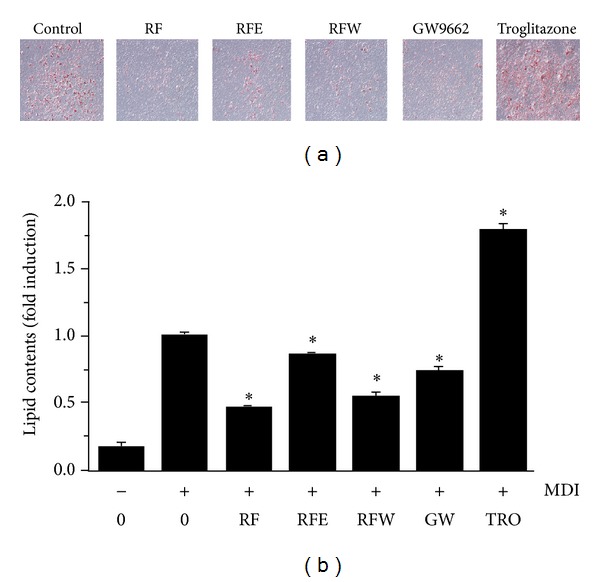
Effect of RF fraction on lipid accumulation of 3T3-L1 adipocyte differentiation. Postconfluent 3T3-L1 cells were differentiated in the absence or presence of RF, RF ethyl acetate fraction (RFE), and RF water fraction (RFW) for 6 days. (a) Lipid droplets were measured by Oil Red O staining. (b) Lipid content was quantified by measuring absorbance. Troglitazone was used as positive control, and GW9662 was used as negative control. Data are expressed as the means ± S.D. where *P* < 0.05 was considered a statistically significant difference from the differentiated control.

To determine the comparative effects of RF extraction fraction, total water-soluble extract was sequentially extracted with ethyl acetate (EtOAC fraction, RFE) and water (water fraction, RFW). The 3T3-L1 cells were treated with three extracts together with MDI, and then the extent of adipocyte differentiation was determined by the Oil Red O staining method. As shown in Figures 2(a) and 2(b), among the three extracts, total water-soluble extract of RF (RF) was most effective in inhibiting adipocyte differentiation indicating that antiadipogenic activities might have been enriched RF. Troglitazone, one of the antidiabetic drugs and a ligand for PPAR*γ*, was used as a positive control, and 2-chloro-5-nitro-N-phenylbenzamide GW9662, an antagonist for PPAR*γ*, was used as a negative control.

### 3.3. Effect of RF on the Expression of PPAR*γ* and C/EBP*α* in 3T3-L1 Adipocytes

**Figure 3:**
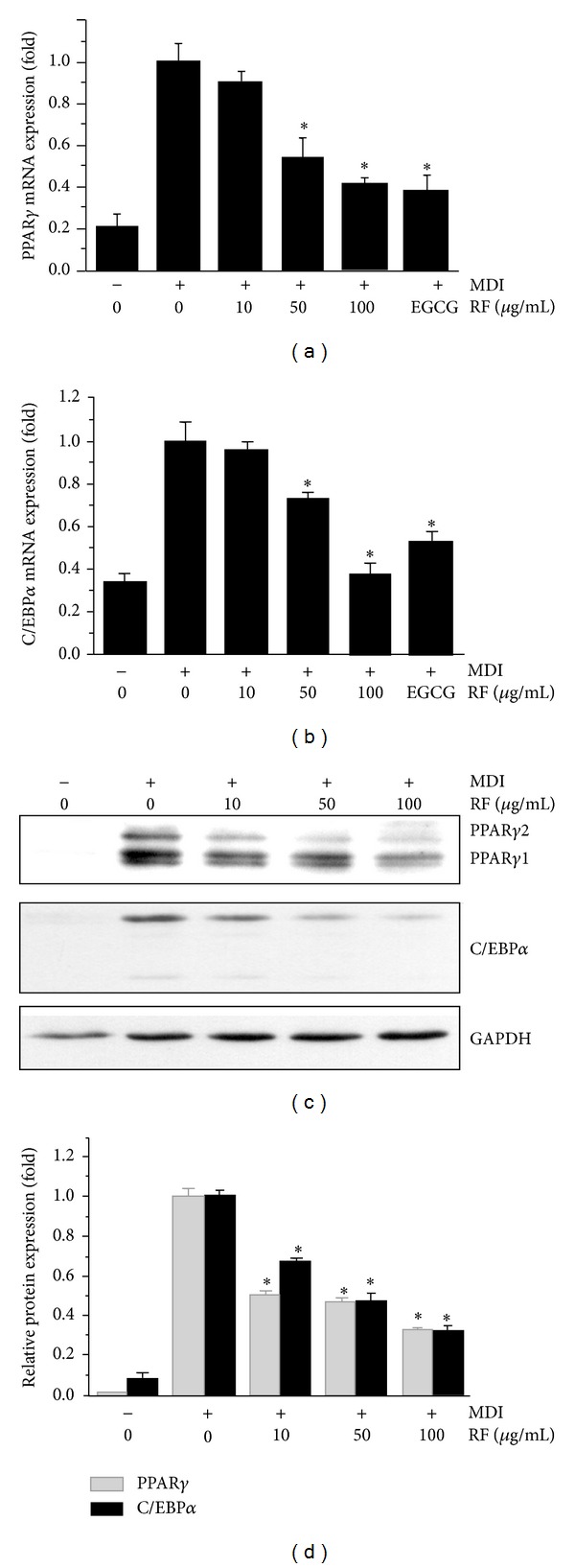
Effect of RF on the expression of PPAR*γ* and C/EBP*α* in 3T3-L1 cells. Postconfluent 3T3-L1 cells were differentiated in the absence or presence of RF (0, 10, 50, and 100 *μ*g/mL) for 6 days. (a) PPAR*γ* and (b) C/EBP*α* mRNA expressions were evaluated by quantitative real-time RT-PCR. (c) PPAR*γ* and (d) C/EBP*α* protein expressions were analyzed by Western blot analysis. Data are expressed as the means ± S.D. where *P* < 0.05 was considered a statistically significant difference from the differentiated control.

To investigate whether RF suppresses adipogenesis through a PPAR**γ** pathway, gene expressions of PPAR*γ* and C/EBP*α* were evaluated by quantitative real-time RT-PCR and Western blot analysis, respectively, after the treatment of fully differentiated cells with 10–100 *μ*g/mL of RF. We observed that the expressions of PPAR*γ* and C/EBP*α* were strongly inhibited by RF at the mRNA level (Figures 3(a) and 3(b)). We also demonstrated that RF treatment resulted in a dose-dependent suppression of PPAR*γ* and C/EBP*α* at the protein level. PPAR*γ* and C/EBP*α* protein levels were reduced up to 68% by treatment with 100 *μ*g/mL of RF (Figures 3(c) and 3(d)).

### 3.4. Effect of RF on Expression of aP2, Resistin, and Adiponectin in 3T3-L1 Adiposity

**Figure 4:**
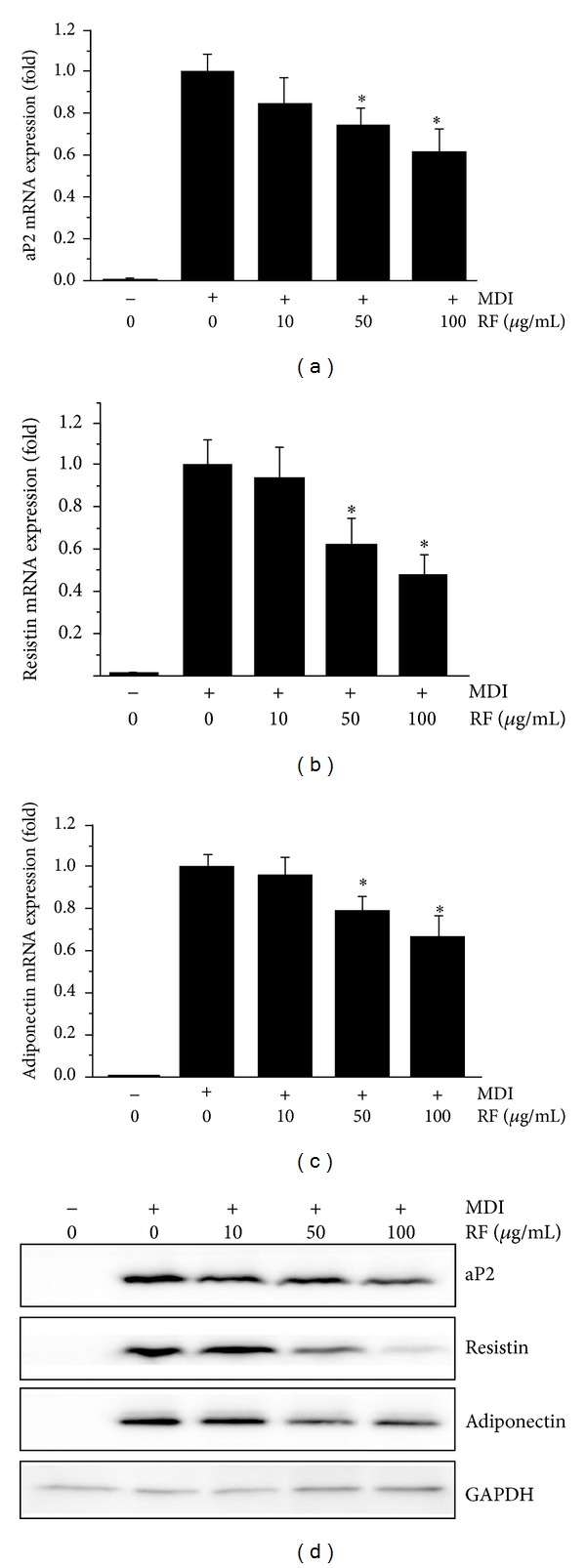
Effect of RF on the expression of aP2, resistin, and adiponectin in 3T3-L1 cells. Postconfluent 3T3-L1 cells were differentiated in the absence or presence of RF (0, 10, 50, and 100 *μ*g/mL) for 6 days. (a) aP2, (b) resistin, and (c) adiponectin mRNA expression were evaluated by the quantitative real-time PCR. (d) aP2, resistin, and adiponectin protein levels were analyzed by Western blot analysis. GAPDH was used as internal controls. Data are expressed as means ± S.D. where *P* < 0.05 was considered a statistically significant difference from the differentiated control.

 We next examined the effects of RF on the expression of adipogenic genes such as aP2, resistin, and adiponectin in 3T3-L1 cells. Fully differentiated cells were treated with 50 or 100 *μ*g/mL of RF, followed by extraction of total RNA for analysis using quantitative real-time PCR. RF treatment with 0.05 mg/mL or 0.1 mg/mL significantly decreased the expression of aP2, resistin, and adiponectin (Figures 4(a), 4(b), and 4(c)). In particular, the resistin mRNA level was reduced up to 53% by treatment with 100 *μ*g/mL of RF (Figure 4(b)). Consistent with the mRNA results, Western blot analysis revealed that RF markedly reduced the protein levels of aP2, resistin, and adiponectin (Figure 4(d)). These results suggest that RF effectively inhibited adipocyte differentiation through the downregulation of adipogenic genes.

### 3.5. The Effect of RF and AMPK siRNA Pretreatment on the Expression and Phosphorylation of Proteins Related to Adipogenesis

**Figure 5:**
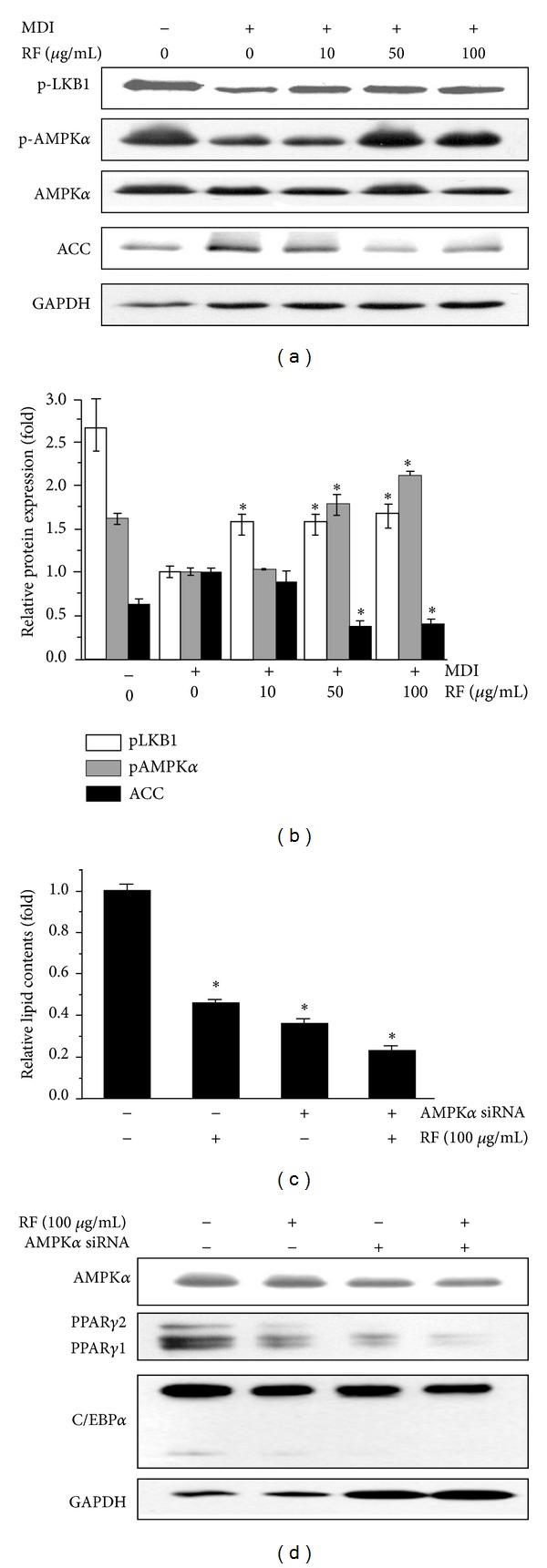
Effects of RF on the phosphorylation of AMPK during 3T3-L1 differentiation. 3T3-L1 preadipocytes were differentiated in the presence of RF (0, 10, 50, and 100 *μ*g/mL) for 6 days. (a) p-LKB1, p-AMPK, and ACC protein levels were analyzed by Western blot analysis. (b) Densitometry analyses were presented as relative ratios of p-LKB1/GAPDH, p-AMPK/AMPK, and ACC/GAPDH. (c) 3T3-L1 cells were treated with RF (Day 4) in AMPK*α* siRNA pretreatment (Day 0). Cells were stained with Oil Red O at 6 days, and lipid contents were quantified. These experiments were conducted as independent experiments in triplicate. Data represent the mean ± S.D. where *P* < 0.05 was considered a statistically significant difference from the differentiated control. (d) After AMPK*α* siRNA pretreatment and RF-treated differentiation, the protein expressions of AMPK*α*, PPAR*γ*, and C/EBP*α* were determined by Western blot analysis.

LKB1, which is an upstream kinase of AMPK, activates AMPK protein in adipose tissue [23]. To investigate whether AMPK, a key player in energy homeostasis [7], is activated by RF during 3T3-L1 differentiation, the protein levels of p-LKB1, p-AMPK*α*, and ACC were analyzed. When compared with the control group, LKB1 and AMPK*α* phosphorylation was increased by treatment with RF. The observed increase in the phosphorylation of LKB1 by RF suggests that RF might upregulate AMPK activity via LKB1. However, the expression of ACC, a downstream target protein of AMPK, was significantly suppressed (Figures 5(a) and 5(b)). To further confirm the above results, adipocytes were pretreated with AMPK*α* siRNA and then treated with 100 *μ*g/mL of RF, and the relative intracellular fat content of each group was determined. AMPK*α* siRNA pretreatment effectively decreased the relative intracellular fat content when compared with the control group suggesting that AMPK*α* siRNA can inhibit adipocyte differentiation with or without RF treatment (Figure 5(c)).  Figure 5(d) shows that the AMPK*α* expression was decreased at the protein level after the siRNA treatment. In addition, both RF and AMPK*α* siRNA treatments decreased the expression of PPAR*γ* and C/EBP*α* proteins.

## 4. Discussion

Obesity is caused by the accumulation of lipid through adipogenesis in adipose tissue, and adipogenesis is the process by which undifferentiated precursor cells differentiate into fat storage cells. Numerous studies have demonstrated that adipocyte differentiation and the amount of fat accumulation are associated with the occurrence and development of obesity. Therefore, the inhibition of adipocyte differentiation is one of the strategies for the treatment of obesity. Recent studies have attempted to investigate the beneficial effects of natural products on obesity. The present study demonstrates the novel effect of RF extract on the inhibition of adipocyte differentiation. Our results show that RF did not cause significant cytotoxic effects in 3T3-L1 cells and significantly inhibited lipid accumulation and adipocyte differentiation in a concentration-dependent manner. These results indicate that RF inhibited adipogenesis during adipocyte differentiation and may have potential antiobesity effects (Figure 1). We also demonstrated that treatment with total water-soluble extract of RF strongly inhibited adipogenesis during adipocyte differentiation relative to an ethyl acetate-soluble fraction of RF (RFE) or water fraction of RF (RFW) (Figure 2).

PPAR*γ* and C/EBP*α* are known to play a role in fat cell function and adipocyte differentiation of preadipocytes [24]. PPAR*γ* induces C/EBP*α* and also increases its own expression. Similarly, C/EBP*α* induces PPAR*γ* expression as well as its own expression. These cooperative functions help in maintaining high levels of PPAR*γ* and C/EBP*α*, and then PPAR*γ* stimulates adipocyte differentiation. Rosen et al. [25] reported that C/EBP*α* can support adipocyte-specific gene expression in the presence of PPAR*γ* at the level of cell morphology and lipid accumulation. In this study, we investigated whether RF can inhibit adipocyte differentiation through the suppression of related transcription factors such as PPAR*γ*- and C/EBP*α*. As a result, RF treatment resulted in reduced expression of PPAR*γ* and C/EBP*α* at both the mRNA and protein levels in 3T3-L1 cells (Figure 3). These results indicate that RF suppresses adipocyte differentiation through a PPAR*γ* and C/EBP*α*-mediated adipogenesis mechanism. We also observed that 3T3-L1 cells treated with RF showed a decreased protein expression as well as mRNA expression of several adipogenesis-related genes including aP2, resistin, and adiponectin, in a dose-dependent manner (Figure 4). A member of the cytoplasmic fatty acid-binding protein family, aP2, was detected in adipose tissue, and its expression was highly regulated during the differentiation of adipocytes [26]. PPAR*γ* binding is required for the function of the fat-selective enhancer for the aP2 gene, in cultured fat cells [24]. Resistin, an adipocyte-secreted molecule, serves as a critical link between obesity and insulin resistance and plays a role in the regulation of glucose homeostasis and hepatic glucose production [27, 28]. Acute administration of recombinant resistin to rats results in impaired glucose tolerance and hepatic insulin resistance [28]. Previous studies have demonstrated that compounds with antiobesity activity inhibit adipocyte differentiation in 3T3-L1 cells through the downregulation of PPAR*γ*, C/EBP*α*, aP2, and resistin [29, 30]. Adiponectin is an adipocyte-derived hormone that plays a role in insulin sensitivity and energy homeostasis [31]. Previous studies have demonstrated that PPAR*γ* agonists can induce an increase in adiponectin levels in 3T3-L1 adipocytes, and that this effect is associated with adipocyte differentiation *via *PPAR response element [32, 33]. 

AMPK, a central sensor of cellular energy, is a eukaryotic heterotrimeric serine/threonine kinase, and it has emerged as a therapeutic target for metabolic disorders including obesity. The activation of AMPK is essential for the inhibition of 3T3-L1 adipocyte lipogenesis by phytochemicals [34]. To determine whether RF inhibits adipocyte differentiation by activating AMPK, the levels of LKB1/AMPK phosphorylation were determined. The results show that the levels of LKB1/AMPK phosphorylation are elevated significantly after RF treatment (Figures 5(a) and 5(b)). Furthermore, RF treatment inactivates the downstream substrate ACC, which is a key enzyme of lipogenesis. This result indicates RF inhibited adipocyte differentiation via the activation of LKB1/AMPK. It was reported that AMPK regulates PPAR*γ* and C/EBP*α*, which are the critical regulators of adipogenesis and fat accumulation in adipocytes [35]. For example, AMPK activator A-769662 inhibits adipocyte differentiation by downregulating PPAR*γ*, C/EBP*α*, FAS, and aP2 [6]. It has been also reported that the treatment of 3T3-L1 preadipocytes with an AMPK activator, AICAR, could inhibit the differentiation process [36]. 

Conversely, Compound C, which is an inhibitor of AMPK, significantly inhibited the adipogenic differentiation of 3T3-L1 cells in a dose-dependent manner, and this inhibitory effect was primarily effective in the initial stage of differentiation [37, 38]. The fact that AMPK signaling might be associated with an adipocyte differentiation program is still controversial, but we found that preadipocyte 3T3-L1 cells were not able to develop to mature adipocytes in the presence of RF and that this effect was promoted when the cells were pretreated with AMPK siRNA (Figure 5(c)). In addition, the expression of PPAR*γ* and C/EBP*α* was markedly decreased by AMPK*α* siRNA pretreatment (Figure 5(d)). Although the downregulation of p-AMPK decreased the protein levels of PPAR*γ* and C/EBP*α*, a direct correlation has not been understood. It has been also reported that AICAR was able to inhibit differentiation either at early or late stages of differentiation [36]. We therefore infer that RF is able to inhibit differentiation with an increasing level of AMPK activity at the late stage of differentiation and not the early stage of differentiation. 

## 5. Conclusion

In conclusion, the objective of this study was to elucidate the effect of RF on adipogenesis in 3T3-L1 cells. This study demonstrated that the extract of RF inhibited adipocyte differentiation of 3T3L-1 and fat accumulation. The antiadipogenic mechanism of RF involves the downregulation of the adipogenic transcription factors, PPAR*γ* and C/EBP*α*, which are related to the expression of aP2, resistin, and adiponectin. In addition, we observed that RF increases AMPK phosphorylation, which plays major roles in the expression of PPAR*γ* and C/EBP*α*. These findings suggest that RF might have a therapeutic effect in the prevention of adipogenesis-related obesity and may be a potential natural drug candidate for the treatment of obesity.

## Figures and Tables

**Table 1:** The primer sequences used for real-time PCR.

Target gene	Primer sequences
PPAR*γ*	5′-TTTTCAAGGGTGCCAGTTTC-3′ (sense)
	5′-TTATTCATCAGGGAGGCCAG-3′ (antisense)
C/EBP*α*	5′-GCCGAGATAAAGCCAAACAA-3′ (sense)
	5′-CCTTGACCAAGGAGCTCTCA-3′ (antisense)
aP2	5′-CGTAAATGGGGATTTGGTCA-3′ (sense)
	5′-TCGACTTTCCATCCCACTTC-3′ (antisense)
Adiponectin	5′-AGACCTGGCCACTTTCTCCTCATT-3′ (sense)
	5′-AGAGGAACAGGAGAGCTTGCAACA-3′ (antisense)
Resistin	5′-TTCCTTGTCCCTGAACTGCT-3′ (sense)
	5′-AGCTCAAGACTGCTGTGCCT-3′ (antisense)
GAPDH	5′-AACTTTGGCATTGTGGAAGG-3′ (sense)
	5′-GGATGCAGGGATGATGTTCT-3′ (antisense)

PPAR*γ*: peroxisome proliferator-activated receptor *γ*; C/EBP*α*: CCAAT-enhancer-binding protein *α*; adipocyte fatty acid-binding protein 2; GAPDH: Glyceraldehyde-3-phosphate dehydrogenase.
